# Alpha-Adducin Gly460Trp Polymorphism and Hypertension Risk: A Meta-Analysis of 22 Studies Including 14303 Cases and 15961 Controls

**DOI:** 10.1371/journal.pone.0013057

**Published:** 2010-09-28

**Authors:** Kuo Liu, Jielin Liu, Yan Huang, Ya Liu, Yuqing Lou, Zuoguang Wang, Hong Zhang, Shan Yan, Zhizhong Li, Shaojun Wen

**Affiliations:** 1 Department of Hypertension Research, Beijing Anzhen Hospital, Capital Medical University and Beijing Institute of Heart Lung and Blood Vessel Diseases, Beijing, People's Republic of China; 2 State Key Laboratory of Genetic Engineering, Institute of Genetics, School of Life Sciences, Fudan University, Shanghai, People's Republic of China; 3 Department of Cardiac Surgery, Beijing Anzhen Hospital, Capital Medical University, Beijing, People's Republic of China; 4 Emergency Center of Heart, Lung and Blood Vessel Diseases, Beijing Anzhen Hospital, Capital Medical University, Beijing, People's Republic of China; Ohio State University Medical Center, United States of America

## Abstract

**Background:**

No clear consensus has been reached on the alpha-adducin polymorphism (Gly460Trp) and essential hypertension risk. We performed a meta-analysis in an effort to systematically summarize the possible association.

**Methodology/Principal Findings:**

Studies were identified by searching MEDLINE and EMBASE databases complemented with perusal of bibliographies of retrieved articles and correspondence with original authors. The fixed-effects model and the random-effects model were applied for dichotomous outcomes to combine the results of the individual studies. We selected 22 studies that met the inclusion criteria including a total of 14303 hypertensive patients and 15961 normotensive controls. Overall, the 460Trp allele showed no statistically significant association with hypertension risk compared to Gly460 allele (P = 0.69, OR = 1.02, 95% CI 0.94–1.10, P_heterogeneity_<0.0001) in all subjects. Meta-analysis under other genetic contrasts still did not reveal any significant association in all subjects, Caucasians, East Asians and others. The results were similar but heterogeneity did not persist when sensitivity analyses were limited to these studies.

**Conclusions/Significance:**

Our meta-analysis failed to provide evidence for the genetic association of α-adducin gene Gly460Trp polymorphism with hypertension. Further studies investigating the effect of genetic networks, environmental factors, individual biological characteristics and their mutual interactions are needed to elucidate the possible mechanism for hypertension in humans.

## Introduction

Essential hypertension (EH) is a major global public health problem which affects a large proportion of adult population worldwide. The occurrence and development of EH are regarded as a complex multifactorial disorder resulted from genetic and environmental factors, as well as their interactions [1]. Although substantial contribution has been made to unravel the pathophysiological mechanisms of hypertension, the molecular genetics of EH is still an intricate and mysterious field. Approximately, 20% to 60% of the population variability in blood pressure (BP) is genetically determined [2]. As a consequence, it is necessary to explore etiology from many of the genes susceptible to hypertension.

Adducin is a ubiquitously expressed heterodimeric cytoskeleton protein composed of two subunits (α-subunit and either β- or γ-subunit) which are encoded by three genes (ADD1, ADD2 and ADD3, respectively) located on different chromosomes [3]. Previous studies in the Milan hypertensive rat strain model of hypertension and humans proved that an altered adducin function might cause hypertension through an enhanced constitutive tubular sodium reabsorption [4]. At the molecular level, the role of alpha-adducin (ADD1) in hypertension and other cardiovascular diseases has been extensively evaluated, paying particular attention to the rs4961 (Gly460Trp, G460W or G460T) single nucleotide polymorphism (SNP) at exon 10 on chromosome 4p16.3 [5], in which a guanine-to-thymine transversion at nucleotide 614 leads to a glycine (Gly) to tryptophan (Trp) substitution at amino acid position 460. In clinical studies, some have reproduced the supportive association between Gly460Trp polymorphism and EH or blood pressure (BP) level [6]–[9], whereas others were unable to replicate these findings [10]–[14]. Although a series of relevant research were carried out on the relationship of this polymorphism to hypertension risk across different nations, the results were still fairly confusing rather than conclusive and showed strong racial and regional variations. Given the accumulation of data, we have conducted a formal meta-analysis from all eligible case-control studies published to date, in order to clarify the role of Gly460Trp polymorphism in hypertension.

## Materials and Methods

### Identification and eligibility of relevant studies

To identify all studies that examined the association of alpha-adducin Gly460Trp polymorphism with hypertension, we conducted a systematic computerized literature search of PubMed and EMBASE databases (up to May 2010) using the following various combinations of keywords and subject terms: ‘adducin’ OR ‘ADD’, ‘polymorphism’ AND ‘hypertension’. We also retrieved additional studies through the MEDLINE option ‘related articles’. Search results were limited to articles in English and studies on human subjects without country restrictions. Only research articles were included. When studies from the same research group with overlapped population were found, only the one with larger population was included to avoid data duplication. The full text of the retrieved articles was scrutinized to decide whether information on the topic of interest was included. References of the retrieved publications were screened. If an article reported results on different ethnicity subpopulations, each subpopulation was treated as a separate study in our meta-analysis. Studies included in the meta-analysis had to meet all the following criteria: (a) using an unrelated case–control design (a retrospective, cross-sectional, or prospective study design), (b) have available genotype frequency, (c) genotype distribution of control population must be consistent with Hardy–Weinberg equilibrium (HWE). Hypertension was defined as systolic BP (SBP) ≥140 mmHg and/or diastolic BP (DBP) ≥90 mmHg and/or treatment with anti-hypertensive medication.

### Data extraction

In order to extract the information needed, all articles were reviewed and separately collated by two independent investigators (K.L., J.L.) who checked for any discordance and reached a consensus. If they could not come to an agreement, a third one adjudicated the disagreements. The following information was collected on the genotype of Gly460Trp according to different kinds of ethnicities. First author, year of publication, country and racial descent of the subjects included, study design and characteristics were described in Table S1. Diagnostic standard of each study, sample sizes of cases and controls, genotype numbers, allele frequency in both cases and controls, and P values of HWE in controls were summarized in Table S2.

### Statistical analysis

The strength of the association of Gly460Trp with hypertension was measured by odds ratio (OR) corresponding to 95% confidence interval (CI) which was calculated according to the method of Woolf [15]. We examined the association between Trp allele of alpha-adducin Gly460Trp and hypertension risk (Trp vs. Gly), the dominant genetic model (GlyTrp+TrpTrp vs. GlyGly), the recessive genetic model (TrpTrp vs. GlyTrp+GlyGly), and homozygote comparison (TrpTrp vs. GlyGly). In our study, two models of meta-analysis were applied for dichotomous outcomes in Review-Manager 4.2 software: the fixed-effects model and the random-effects model. The fixed-effects model using the Mantel–Haenszel method, assumes that studies are sampled from populations with the same effect size, making an adjustment to the study weights according to the in-study variance. The random-effects model using the DerSimonian and Laird's method, which assumes that studies are taken from populations with varying effect sizes, calculating the study weights both from in-study and between-study variances, considering the extent of variation, or heterogeneity. We performed a chi-square-based Q statistic test to assess the between-study heterogeneity [16]. Heterogeneity was considered significant for p<0.10 because of the low power of the statistic. The inconsistency index I^2^ was also calculated to evaluate the variation which was caused by heterogeneity rather than by chance, and higher values of the index indicate the existence of heterogeneity [17]. The fixed-effects model (if p>0.10) or the random-effects model (if p<0.10) was used to pool the results [18]. The significance of the pooled OR was determined by the Z test and a P value of <0.05 was considered significant. Studies were also categorized into subgroups based on ethnicity. For each genetic comparison, subgroup analyses were considered for the population of Caucasians, East Asians and others to estimate ethnic-specific OR. The subgroup ‘others’ included the South African Negro population and African-American population. In addition, in order to further uncover the underlying role of the genetic variation in ADD1, subgroup analyses on different diagnostic standards for hypertension were also carried out.

Sensitivity analyses were conducted by sequential deleting a single study each time in an attempt to identify the potential influence of the individual data set to the pooled ORs. In addition, an estimate of potential publication bias was carried out by the funnel plot, in which the standard error of log (OR) of each study was plotted against its OR. An asymmetric plot suggested possible publication bias. Funnel-plot asymmetry was assessed by the method of Egger's linear regression test [19]. Furthermore, we performed a T-test to determine the significance of the intercept, and a P value lower than 0.05 was considered to be statistically significant. HWE was tested by the chi-square test for goodness of fit based on a Web program (http://www.ihg.gsf.de/cgi-bin/hw/hwa1.pl).

Analyses were performed using the software Review-Manager 4.2 (Oxford, England) and Stata version 10.0 (Stata Corporation, College Station, Texas, USA). All P-values were two-sided.

## Results

### Description of studies identified in meta-analysis

**Figure 1:**
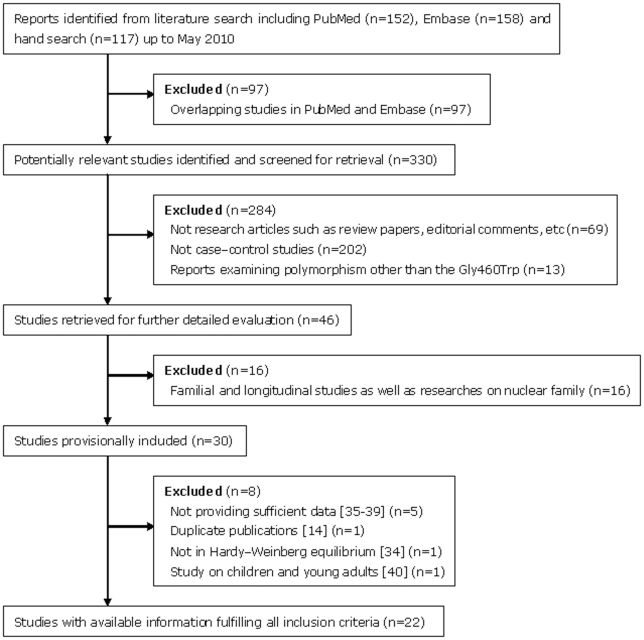
Flow chart of selection of studies and specific reasons for exclusion from the meta-analysis.

The initial search strategy for hypertension susceptibility related to the alpha-adducin SNP yielded 152 potentially relevant references in PubMed and 158 in EMBASE, most of which were overlapping. In addition, we supplemented the search with a hand search of reference lists from published literature (117 articles). After the subsequent screening, 30 studies were identified for recruitment in the light of the inclusion criteria [6]–[14], [20]–[40]. All studies were published between 1997 and 2010. Among the 30 eligible articles, the study of He et al. [14] was replaced by their latter report [33] which including larger population. Samples selected were all men (both cases and controls) in Psaty et al. [22] Only one paper by Province et al. [23] included separate data on subjects of two ethnicities: European-American and African-American, however, the genotyping data in the population of European-American was deviated from HWE (P_HWE_ = 0.000522), as well as in the population of Asian provided in Ramachandran et al. [34] (P_HWE_ = 0.001644). Thus, those two studies were excluded. Seven papers did not provide relevant data or the data provided were not sufficient, we have contacted corresponding or original authors by e-mail in order to obtain the raw data. Two of them have replied and offered the necessary data [25], [32], and the other five have not yet responded [35]–[39]. Furthermore, we also deleted an article by Marcun Varda et al. [40] that described hypertension in children and young adults, in which the diagnostic standard for EH is different from adults [41]. In conclusion, 22 studies were selected for inclusion in our final analyses (Table S1). All studies used blood sample for genotyping. The flow chart summarizing the process of study selection and reasons for exclusion is presented in Figure 1.

### Summary statistics

A total of 14303 hypertension patients and 15961 controls were investigated. Population-based and hospital-based studies were all included in our meta-analysis. Twelve studies were population-based [9], [13], [20], [22], [23], [25]–[28], [30]–[32], and ten were hospital-based studies [6]–[8], [10]–[12], [21], [24], [29], [33]. Populations among these studies were as following: ten studies were Caucasians or European descent (7632 cases and 8588 controls), nine studies were East Asians (5957 cases and 6770 controls) and three studies were others (one study recruited South African Negro subjects and the other two studies recruited African-American subjects) involving 714 cases and 603 controls. All genotypes and allele frequencies of cases and controls were shown in Table S2. The pooled overall frequency of the Trp allele were 34.91% and 35.23% in hypertensive cases and in normotensive controls, respectively. Allele Trp had a higher representation in cases and controls of East Asians (56.67% and 55.38%, respectively) than that of Caucasians (20.51% and 21.30%, respectively) and others (7.21% and 7.30%, respectively).

### Main results

**Figure 2:**
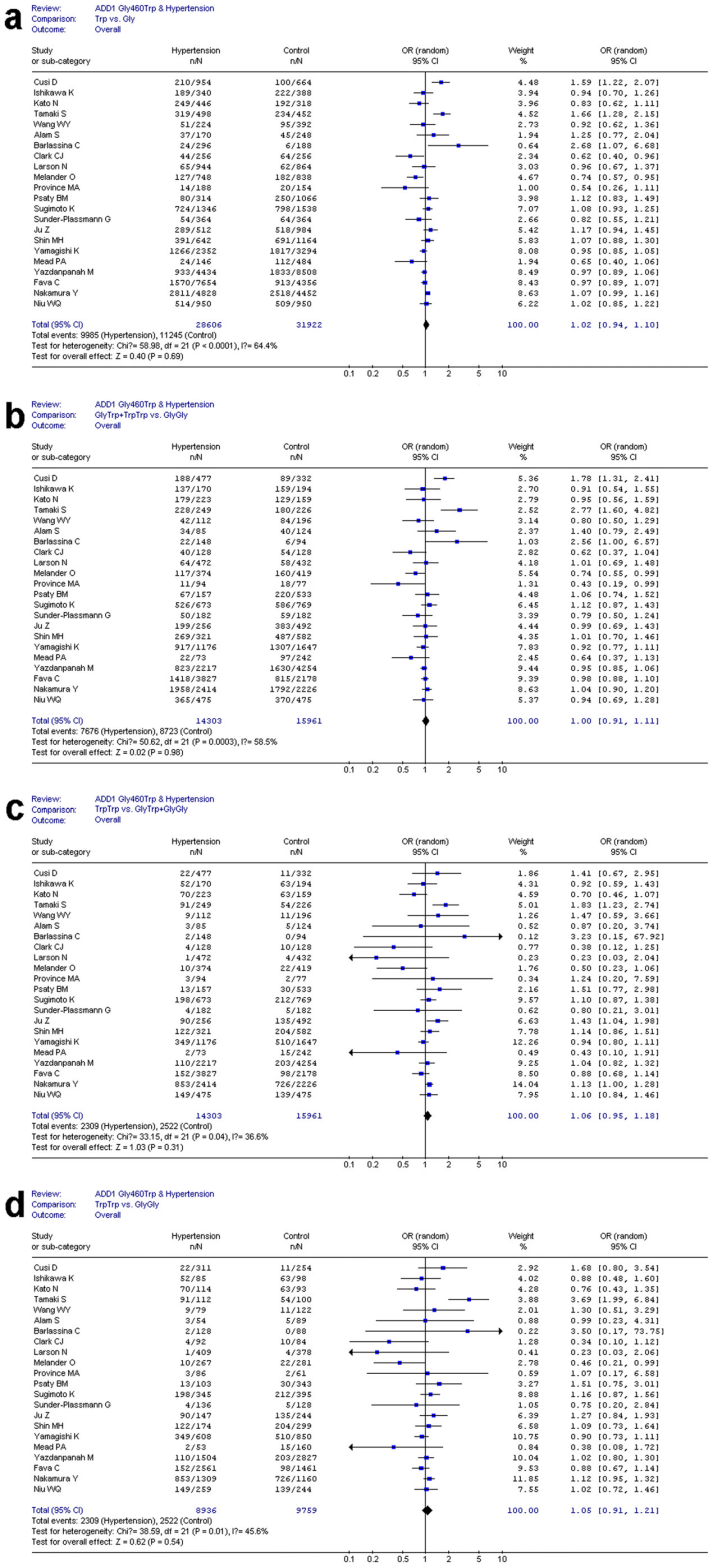
Association between ADD1 Gly460Trp polymorphism and hypertension risk in overall populations under various genetic contrasts. Figure 2a shows the association between Gly460Trp polymorphism and hypertension in allele comparison (Trp vs. Gly). n indicates the total number of Trp allele, N indicates the total number of Trp allele plus Gly allele. Figure 2b shows the association between Gly460Trp polymorphism and hypertension under dominant genetic model (GlyTrp+TrpTrp vs. GlyGly). n indicates the total number of GlyTrp+TrpTrp, N indicates the total number of individuals. Figure 2c shows the association between Gly460Trp polymorphism and hypertension under recessive genetic model (TrpTrp vs. GlyTrp+GlyGly). n indicates the total number of TrpTrp, N indicates the total number of individuals. Figure 2d shows the association between Gly460Trp polymorphism and hypertension in homozygote comparison (TrpTrp vs. GlyGly). n indicates the total number of TrpTrp, N indicates the total number of TrpTrp plus GlyGly.

For each study, we investigated the association between the alpha-adducin Gly460Trp polymorphism and hypertension risk, assuming different inheritance models of the 460Trp allele (Table 1). Overall, when all the eligible studies were pooled into the meta-analysis, no significant associations between ADD1 Gly460Trp polymorphism and hypertension susceptibility were observed in all genetic models. No significant associations were found for allele comparison (Trp vs. Gly: P = 0.69, OR = 1.02, 95% CI 0.94–1.10, P_heterogeneity_<0.0001) (Figure 2a), dominant genetic mode (GlyTrp+TrpTrp vs. GlyGly: P = 0.98, OR = 1.00, 95% CI 0.91–1.11, P_heterogeneity_ = 0.0003) (Figure 2b), recessive genetic model (TrpTrp vs. GlyTrp+GlyGly: P = 0.31, OR = 1.06, 95% CI 0.95–1.18, P_heterogeneity_ = 0.04) (Figure 2c), and homozygote comparison (TrpTrp vs. GlyGly: P = 0.54, OR = 1.05, 95% CI 0.91–1.21, P_heterogeneity_ = 0.01) (Figure 2d). Similarly, no significant association was detected in all genetic models in the subgroup analyses by ethnicity and different diagnostic criteria for hypertension (Table 1).

**Table 1:** Summary estimates for ORs and 95% CI in different subgroups under various genetic contrasts.

Genotype contrasts	Study population	Study numbers	P_heterogeneity_	P*value	OR	95% CI
Allele comparison	Overall	22	<0.0001	0.69^b^	1.02	0.94–1.10
(Trp vs. Gly)	Caucasian	10	0.001	0.51^b^	0.96	0.84–1.09
	East Asian	9	0.01	0.19^b^	1.06	0.97–1.16
	Others	3	0.03	0.90^b^	1.05	0.51–2.14
	SBP≥160, DBP≥100	2	0.31	0.62^a^	0.98	0.90–1.07
	SBP≥160, DBP≥95	7	0.08	0.38^b^	0.94	0.81–1.09
	SBP≥150, DBP≥95	10	<0.00001	0.61^b^	1.05	0.87–1.28
Dominant model	Overall	22	0.0003	0.98^b^	1.00	0.91–1.11
(GlyTrp+TrpTrp vs. GlyGly)	Caucasian	10	0.001	0.56^b^	0.96	0.82–1.11
	East Asian	9	0.06	0.51^b^	1.05	0.91–1.20
	Others	3	0.02	0.98^b^	1.01	0.45–2.25
	SBP≥160, DBP≥100	2	0.19	0.47^a^	0.96	0.87–1.07
	SBP≥160, DBP≥95	7	0.17	0.26^a^	0.95	0.87–1.04
	SBP≥150, DBP≥95	10	<0.00001	0.30^b^	1.14	0.89–1.46
Recessive model	Overall	22	0.04	0.31^b^	1.06	0.95–1.18
(TrpTrp vs. GlyTrp+GlyGly)	Caucasian	10	0.28	0.54^a^	0.95	0.82–1.11
	East Asian	9	0.02	0.13^b^	1.10	0.97–1.25
	Others	3	0.32	0.69^a^	0.80	0.26–2.43
	SBP≥160, DBP≥100	2	0.81	0.76^a^	1.04	0.82–1.31
	SBP≥160, DBP≥95	7	0.29	0.24^a^	0.90	0.75–1.08
	SBP≥150, DBP≥95	10	0.01	0.67^b^	0.94	0.69–1.27
Homozygote comparison	Overall	22	0.01	0.54^b^	1.05	0.91–1.21
(TrpTrp vs. GlyGly)	Caucasian	10	0.16	0.45^a^	0.94	0.80–1.10
	East Asian	9	0.007	0.23^b^	1.12	0.93–1.35
	Others	3	0.33	0.65^a^	0.77	0.25–2.35
	SBP≥160, DBP≥100	2	0.97	0.88^a^	1.02	0.80–1.29
	SBP≥160, DBP≥95	7	0.36	0.33^a^	0.91	0.74–1.10
	SBP≥150, DBP≥95	10	0.0005	0.94^b^	0.98	0.64–1.52

Abbreviations: OR, odds ratio; CI, confidence interval.

a Fixed effect estimate,

b Random effect estimate.

*The P-value of OR determined by the Z test.

### Sensitivity analysis

Sensitivity analyses were conducted to assess whether each individual study affected the final results. The findings revealed that no individual study affected the results in all subjects. In the subgroup analysis, three independent studies (Cusi et al. [6], Tamaki et al. [7], and Barlassina et al. [8]) were considered as the main cause of heterogeneity for Caucasians, East Asians, and others, respectively. The three studies were all hospital-based. After exclusion of these studies, the heterogeneity no longer existed, but still reached a negative association. (data not shown).

### Publication bias

**Figure 3:**
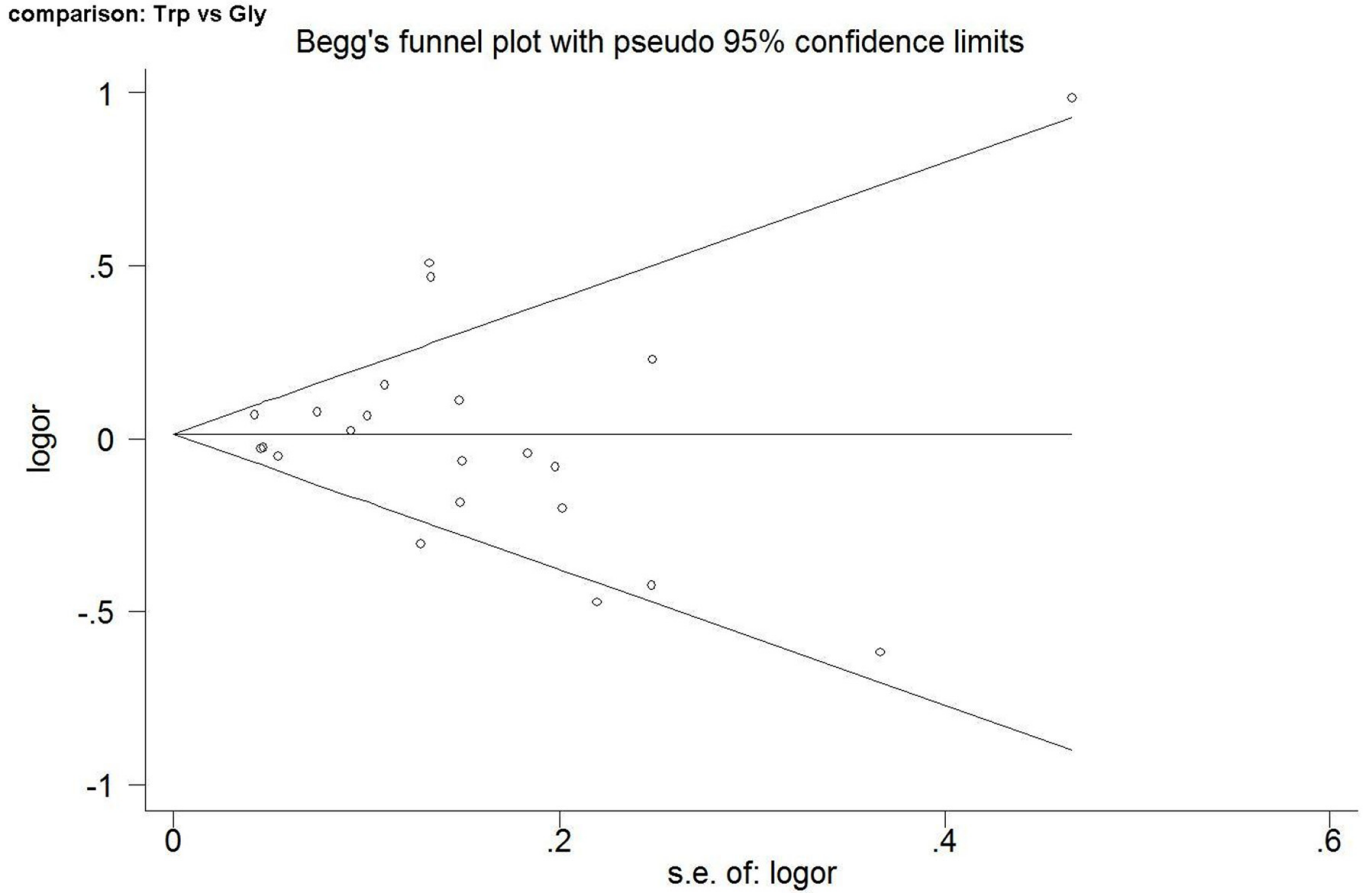
Begg's funnel plot analysis to detect publication bias for allele comparison (Trp vs. Gly). Egger's test was also performed to investigate the symmetry of the funnel plot, and no asymmetry was found as indicated by the P value of Egger's test.

The funnel plot was applied for comparison of 460Trp versus Gly460 in the OR analysis of alpha-adducin Gly460Trp, and Egger's test provided no evidence for funnel-plot asymmetry (t = −0.06, P = 0.954) (Figure 3).

## Discussion

To the best of our knowledge, this is one of the largest systematic review of the literature by means of a meta-analysis to date investigating the association between alpha-adducin Gly460Trp polymorphism and EH. Compared with the recent case-control study and meta-analysis by Niu et al. [33], our meta-analysis conducted here included in total 22 studies with 14303 cases and 15961 controls which were almost three times higher than the study mentioned above. However, we failed to obtain association of Gly460Trp polymorphism with hypertension, even in the stratification analyses based on ethnicity or different diagnostic criteria.

In 1997, Cusi et al. first reported that 460Trp allele of alpha-adducin was associated with hypertension in human subjects, particularly in a salt-sensitive form of EH [6]. Manunta et al. found that the Gly460Trp polymorphism of ADD1 modulates the overall capacity of tubular epithelial cells to transport ions through variations in sodium-potassium ATPase activity and through modifications in the assembly of the actin cytoskeleton [42]. Further findings indicated a reduced renal pressure-natriuresis slope after sodium depletion or sodium load in individuals with Trp allele, which confirmed that humans bearing one Trp allele alpha-adducin variant displayed an increased of renal tubular sodium reabsorption and sodium retention [43]. These findings suggested that 460Trp variant of the α-adducin was probably associated with a sodium-sensitive form of hypertension, which was caused by physiology or pathophysiology mechanisms mentioned above. However, the results of our meta-analysis showed no significant association between Gly460Trp polymorphism and hypertension. It seemed that 460Trp variant was associated with renal Na^+^ handling (sodium transport and reabsorption) rather than the salt-sensitive form of EH. Among white hypertensive patients in Italy, five salt sensitivity studies conducted by BP response to acute salt loading test (saline infusion) and chronic diuretic treatment effect were performed by the same research group, which showed evidence to support the role of 460Trp in salt-related hypertension [6], [43]–[46]. Grant et al. replicated the above result, using a high and low sodium diet [47], while another study failed to detect this effect in Polish hypertensives [48]. Turner et al. reported a negative study involved two ethnicities (African American and non-Hispanic white), revealing that no relationship existed between α-adducin genotypes and the BP response to the diuretics [49]. A recent study by Suonsyrjä et al. showed no influence of Trp allele on the magnitude of BP fall with diuretics among Finnish hypertensive men [50]. Taken together with previous results, the impact of 460Trp on diuretics treatment varied among different ethnic and geographic populations. Results from a newly published meta-analysis investigating the association between α-adducin gene polymorphism (Gly460Trp) and genetic predisposition to salt sensitivity suggested that Gly460Trp polymorphism in general is not significantly associated with salt-sensitivity (P = 0.08, OR = 1.40, 95% CI 0.96–2.04) [51]. This was consistent with our results. However, considering the potential confounders of studies included in the meta-analysis, our results should be interpreted with caution.

Furthermore, it is important to highlight that, EH is a complex polygenic disease responsive to environmental factors. A single polymorphism or gene likely has weak effects on the individual's phenotype, because multiple genes and genetic interactions have been implicated in the regulation of BP. A family study from van Rijn et al. [52] showed that the Gly460Trp polymorphism of ADD1 was able to explain only a very small proportion of the heritability of BP traits (approximately 0.2% of the SBP variance, P = 0.16; 0.1% of DBP variance, P = 0.78; 0.3% of the variance of pulse pressure, P = 0.07). Therefore, it is necessary to explore the combined effect of Gly460Trp with other related polymorphisms in the same gene or different genes such as ADD2, ADD3, and angiotensin-converting enzyme gene (ACE). Lanzani et al. provided evidence for an epistatic interaction of ADD1 and ADD3 gene variants was associated with variation in BP among the hypertensive patients [53]. Another similar study by Staessen et al. reported that epistatic interaction between the ACE and ADD1 contributed to the prevalence and incidence of hypertension in Caucasians [54]. Epistasis is the potentiation or suppression of a gene by other non-allelic genes [55], which is probably ignored in the majority of case-control and population studies. Consequently, the negative findings of studies on the genetic determinants of EH focused on single-gene effect are not surprising in these settings. More studies based on larger population and well-designed, especially studies investigating the combined effect of Gly460Trp and other polymorphisms are required to further evaluate the role of these polymorphisms in hypertension.

Moreover, environmental factors and individual biological characteristics ought not to be neglected. The former include, most importantly, salt intake, smoking, alcohol consumption, etc. For instance, Yamagishi et al. pointed out that the α-adducin TrpTrp genotype was associated with higher systolic BP among men with a higher sodium intake [27]. The latter include race, age, gender, body mass index (BMI), health condition, and general physiological/neurological functioning, etc. Perhaps most important among above factors is age, which is a determinant of the penetrance of genetic variants [56]. Older age increases Na^+^ sensitivity, makes the relationship between BP and exchangeable body Na^+^ stronger, reduces renal perfusion, and compromises the buffering effects of the large arteries on both systolic and diastolic pressure [56]. On these grounds, comprehensive consideration of interrelations and interactions with the context of genetic backgrounds, environmental factors, and individual biological characteristics is indispensable when exploring the association between a single gene variant and hypertension risk. Just as Bianchi et al. mentioned that ‘when context is taken into account, the impact of adducin in hypertension and its related disorders is clear’ [57], [58].

**Figure 4:**
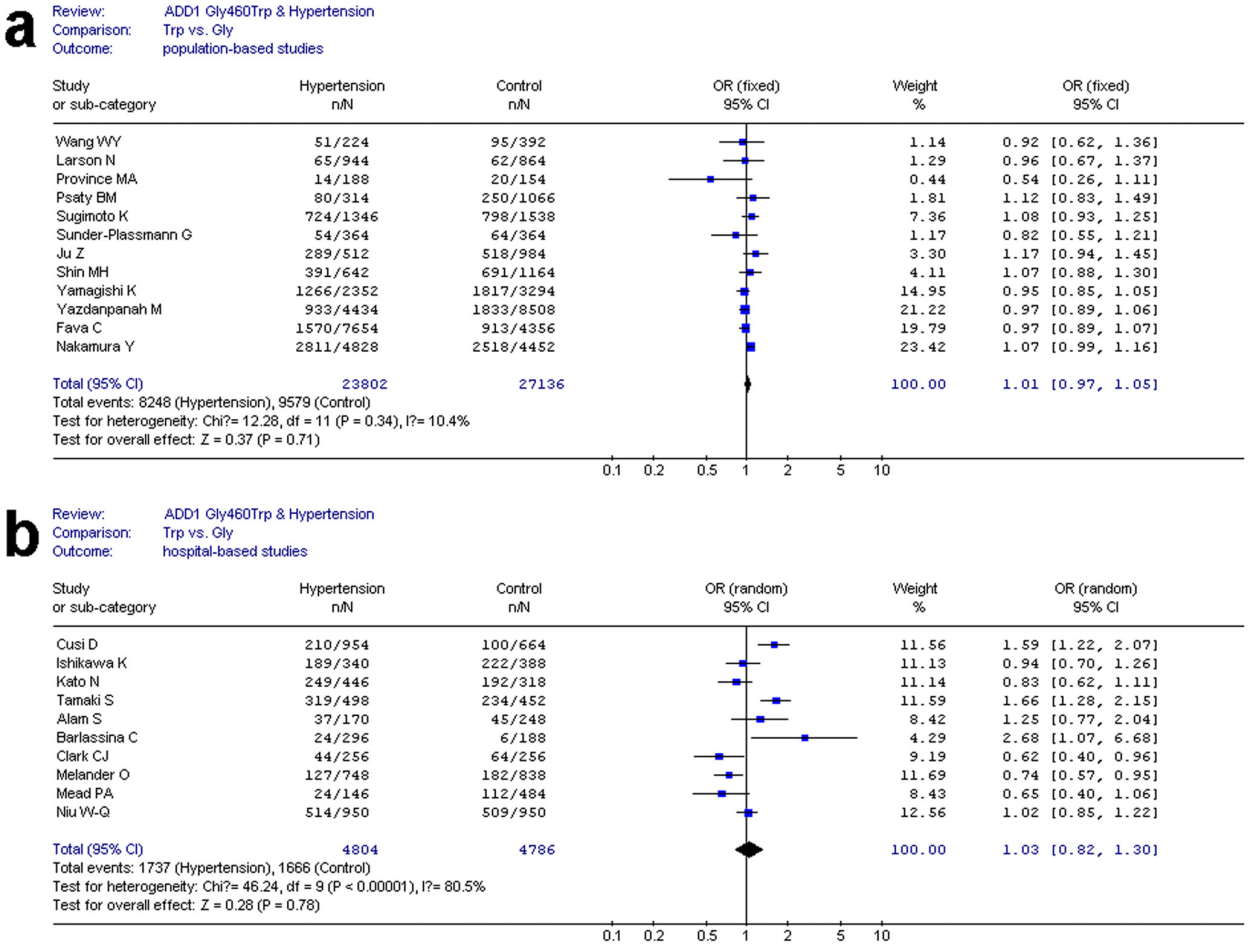
Forest plot of Gly460Trp polymorphism and hypertension risk on different sample sources for allele comparison. Figure 4a shows the forest plot of ADD1 Gly460Trp polymorphism and hypertension risk among population-based studies in allele comparison. Figure 4b shows the forest plot of ADD1 Gly460Trp polymorphism and hypertension risk among hospital-based studies in allele comparison.

In the sensitivity analysis, three hospital-based studies (Cusi et al. [6], Tamaki et al. [7], and Barlassina et al. [8]) were considered as the main cause of heterogeneity. In order to uncover the potential influence of the sample sources (population or hospital-based), further research was conducted on the different sources of subjects. The results indicated that no heterogeneity was observed in population-based stuides (P_heterogeneity_ = 0.34, I^2^ = 10.4%, allele comparison) (Figure 4a), while considerable heterogeneity existed in hospital-based stuides (P_heterogeneity_<0.00001, I^2^ = 80.5%, allele comparison) (Figure 4b). There were still no significant association in the two analyses above. The controls of the hospital-based studies might be ill-related population. They might not be a representative of the general population. Thus, such studies usually have some selection biases, which might affect the quality and reliability of individual studies.

The results of the present meta-analysis should be interpreted within the context of its limitations. First, this meta-analysis only focused on papers published in the English language, so some local literature biases were inevitable. Second, not all the control subjects were age- and sex- matched to cases in the studies included in our meta-analysis, which was likely one of the causes for heterogeneity. In addition, another potential confounding effect is that some of the young normotensive controls might develop hypertension later in their life. Perhaps using stricter criteria for the selection of normal controls, such as normotensive subjects aged 55–60 years or older, could exclude the false-negative controls [59], [60]. Third, the strong heterogeneity observed in the relationship between the Gly460Trp polymorphism and hypertension precluded a consistent estimate of the effect of this variant, even when analyzed by ethnicity. Furthermore, in our study, we did not perform an evaluation of potential interactions such as gene-gene, gene-environment, which might influence the results.

In conclusion, our meta-analysis suggested that the Gly460Trp polymorphism in ADD1 was not associated with susceptibility to hypertension even upon stratification by ethnicity or diagnostic criteria. Based on our discussion and the results from the meta-analysis, we may conclude that the Gly460Trp polymorphism is probably not a genetic indicator for increased risk of hypertension. To better understand the potential mechanism for hypertension in humans, future research should be carried out to explore the effect of genetic networks, environmental factors, individual biological characteristics and their mutual interactions.

## Supporting Information

Table S1 Detailed characteristics of eligible studies considered in the meta-analysis
(0.10 MB DOC)

Table S2 Diagnostic standard of each study, the distribution of Gly460Trp genotypes and alleles among hypertension of cases and controls, and P-values of HWE in controls
(0.09 MB DOC)
