# Supplementary material for: Alpha-Adducin Gly460Trp Polymorphism and Hypertension Risk: A Meta-Analysis of 22 Studies Including 14303 Cases and 15961 Controls
Source: PLoS One. 2010 Sep 28;5(9):e13057. doi: 10.1371/journal.pone.0013057 (PMC2946925; doi:10.1371/journal.pone.0013057)
Supplement: Table S1 — Detailed characteristics of eligible studies considered in the meta-analysis (0.10 MB DOC) [file pone.0013057.s001.doc]

**Table S1 Detailed characteristics of eligible studies considered in the meta-analysis**

|  |  |  |  | Characteristics | |
| --- | --- | --- | --- | --- | --- |
| First author | Year | Country | Ethnicity | Cases | Controls |
| Cusi [6]^1^ | 1997 | Italy | Caucasian | Free of any other disease | Without a family history of hypertension |
|  |  | French |  |  |  |
| Kato [10]^1^ | 1998 | Japan | East Asian | Non-diabetic, secondary form of | Blood pressure values ﹤130/85 mm Hg |
|  |  |  |  | hypertension were excluded |  |
| Tamaki [7]^1^ | 1998 | Japan | East Asian | Patients older than 65 years were excluded | No cardiovascular or other diseases |
| Ishikawa [11]^1^ | 1998 | Japan | East Asian | Patients had a family history of | Age- and sex- matched, no family history of |
|  |  |  |  | hypertension in frist-degree relatives, | hypertension, non-diabetic |
|  |  |  |  | secondary hypertension, diabetes mellitus |  |
|  |  |  |  | were excluded |  |
| Wang [20]^1^ | 1999 | Australia | Caucasian | Treated patients, non-diabetic | Age- and sex- matched, no family history of |
|  |  |  | (British descent) |  | hypertension |
| Melander [21]^1^ | 2000 | Sweden | Caucasian | Any other chronic disease were excluded | No family history of hypertension, no chronic |
|  |  | Finland |  |  | disease |
| Psaty [22]^2^ | 2000 | Netherlands | Caucasian | Hypertensive patients suffering from | Renal disease, severe neuropsychiatric |
|  |  |  |  | myocardial infarction | problems were excluded and age- matched |
| Alam [12]^1^ | 2000 | Australia | Caucasian | Patients with ISH in elderly (≥ 60 years), | No family history of hypertension, DM 6.5% |
|  |  |  | (British descent) | DM 10.3% |  |
| Barlassina [8]^1^ | 2000 | South Africa | African | Free of other cardiovascular disease, | Any other chronic disease were excluded |
|  |  |  |  | non-diabetic |  |
| Larson [13]^1^ | 2000 | USA | African-American | Hypertensive individuals, DM 17.16% | Age- and sex- matched, DM 10.93% |
| Province [23]^1^ | 2000 | USA | African-American | Hypertensive individuals | Normotensive individuals |
| Clark [24]^1^ | 2000 | UK | Caucasian | Patients with positive family history of | Age- and sex- matched |
|  |  |  |  | hypertension |  |
| Sugimoto [25]^1^ | 2002 | Japan | East Asian | Patients older than 40 years, DM 20.2% | Age- and sex- matched, DM 14.0% |
| Sunder- Plassmann [26]^1^ | 2002 | Austria | Caucasian | Patients with hypertensive crisis | Age- and sex- matched healthy individuals |
|  |  |  |  | (DBP≥120 mm Hg) |  |
| Ju [9]^1^ | 2003 | China | East Asian | Non-diabetic, renal disease were excluded | Normotensives were matched for age |
| Yamagishi [27]^2^ | 2004 | Japan | East Asian | Hypertensive individuals | Normotensive individuals |
| Shin [28]^2^ | 2004 | South Korea | East Asian | Hypertensive individuals, DM 6.5% | Normotensive individuals, DM 4.6% |
| Mead [29]^1^ | 2005 | UK | Caucasian | Patients with positive family history of | Normotensive, non-diabetic |
|  |  |  |  | hypertension |  |
| Yazdanpanah [30]^3^ | 2006 | Netherlands | Caucasian | Patients older than 55 years, a total of 599 | Normotensive individuals, a total of 599 DM in |
|  |  |  |  | DM in all the subjects | all the subjects |
| Nakamura [31]^2^ | 2007 | Japan | East Asian | Patients older than 35 years | Normotensive individuals |
| Fava [32]^2^ | 2007 | Sweden | Caucasian | Hypertensive individuals | Normotensive individuals |
| Niu [33]^1^ | 2010 | China | East Asian | Hypertensive individuals | Healthy controls, no familial hypertension |
|  |  |  |  |  | history, free of diabetes and renal disease, |
|  |  |  |  |  | age- and sex- matched |

Abbreviations: ISH, Isolated systolic hypertension ; DM, diabetes mellitus.

^1^ case–control study, ^2^ cross-sectional study, ^3^ Prospective cohort study.
