# Supplementary material for: Alpha-Adducin Gly460Trp Polymorphism and Hypertension Risk: A Meta-Analysis of 22 Studies Including 14303 Cases and 15961 Controls
Source: PLoS One. 2010 Sep 28;5(9):e13057. doi: 10.1371/journal.pone.0013057 (PMC2946925; doi:10.1371/journal.pone.0013057)
Supplement: Table S2 — Diagnostic standard of each study, the distribution of Gly460Trp genotypes and alleles among hypertension of cases and controls, and P-values of HWE in controls (0.09 MB DOC) [file pone.0013057.s002.doc]

**Table S2 Diagnostic standard of each study, the distribution of Gly460Trp genotypes and alleles among hypertension of cases and controls, and P-values of HWE in controls**

|  |  |  |  |  |  |  |  |  |  | T allele | | HWE |
| --- | --- | --- | --- | --- | --- | --- | --- | --- | --- | --- | --- | --- |
|  |  | Number | | GG genotype | | GT genotype | | TT genotype | | frequency (%) | | P^*^ value |
| First author | Diagnostic standard (mmHg) | Cases | Controls | Cases | Controls | Cases | Controls | Cases | Controls | Cases | Controls | Controls |
| Cusi [6] | SBP≥150, DBP≥95 | 477 | 332 | 289 | 243 | 166 | 78 | 22 | 11 | 22.01 | 15.06 | 0.14 |
| Kato [10] | SBP≥160, DBP≥95 | 223 | 159 | 44 | 30 | 109 | 66 | 70 | 63 | 55.83 | 60.38 | 0.09 |
| Tamaki [7] | SBP≥150, DBP≥95 | 249 | 226 | 21 | 46 | 137 | 126 | 91 | 54 | 64.06 | 51.77 | 0.08 |
| Ishikawa [11] | SBP≥160, DBP≥95 | 170 | 194 | 33 | 35 | 85 | 96 | 52 | 63 | 55.59 | 57.22 | 0.88 |
| Wang [20] | NS | 112 | 196 | 70 | 112 | 33 | 73 | 9 | 11 | 22.77 | 24.23 | 0.84 |
| Melander [21] | SBP≥160, DBP≥95 | 374 | 419 | 257 | 259 | 107 | 138 | 10 | 22 | 16.98 | 21.72 | 0.52 |
| Psaty [22] | NS | 157 | 533 | 90 | 313 | 54 | 190 | 13 | 30 | 25.48 | 23.45 | 0.87 |
| Alam [12] | SBP≥160, DBP﹤95 | 85 | 124 | 51 | 84 | 31 | 35 | 3 | 5 | 21.76 | 18.15 | 0.58 |
| Barlassina [8] | DBP≥95 | 148 | 94 | 126 | 88 | 20 | 6 | 2 | 0 | 8.11 | 3.19 | 0.75 |
| Larson [13] | SBP≥160, DBP≥95 | 472 | 432 | 408 | 374 | 63 | 54 | 1 | 4 | 6.89 | 7.18 | 0.20 |
| Province [23] | SBP≥140, DBP≥90 | 94 | 77 | 83 | 59 | 8 | 16 | 3 | 2 | 7.45 | 12.99 | 0.48 |
| Clark [24] | SBP≥160, DBP≥90 | 128 | 128 | 88 | 74 | 36 | 44 | 4 | 10 | 17.19 | 25.00 | 0.35 |
| Sugimoto [25] | SBP≥140, DBP≥90 | 673 | 769 | 147 | 183 | 328 | 374 | 198 | 212 | 53.79 | 51.89 | 0.47 |
| Sunder-Plassmann [26] | NS | 182 | 182 | 132 | 123 | 46 | 54 | 4 | 5 | 14.84 | 17.52 | 0.75 |
| Ju [9] | SBP≥140, DBP≥90 | 256 | 492 | 57 | 109 | 109 | 248 | 90 | 135 | 56.45 | 52.64 | 0.81 |
| Yamagishi [27] | SBP≥140, DBP≥90 | 1176 | 1647 | 259 | 340 | 568 | 797 | 349 | 510 | 53.83 | 55.16 | 0.38 |
| Shin [28] | SBP≥140, DBP≥90 | 321 | 582 | 52 | 95 | 147 | 283 | 122 | 204 | 60.90 | 59.36 | 0.85 |
| Mead [29] | SBP≥140, DBP≥90 | 73 | 242 | 51 | 145 | 20 | 82 | 2 | 15 | 16.44 | 23.14 | 0.46 |
| Yazdanpanah [30] | SBP≥160, DBP≥100 | 2217 | 4254 | 1394 | 2624 | 713 | 1427 | 110 | 203 | 21.04 | 21.54 | 0.61 |
| Nakamura [31] | SBP≥140, DBP≥90 | 2414 | 2226 | 456 | 434 | 1105 | 1066 | 853 | 726 | 58.22 | 56.56 | 0.23 |
| Fava [32] | SBP≥140, DBP≥90 | 3827 | 2178 | 2409 | 1363 | 1266 | 717 | 152 | 98 | 20.51 | 20.96 | 0.76 |
| Niu [33] | SBP≥140, DBP≥90 | 475 | 475 | 110 | 105 | 216 | 231 | 149 | 139 | 54.11 | 53.58 | 0.63 |
|  | Total | 14303 | 15961 | 6627 | 7238 | 5367 | 6201 | 2309 | 2522 | 34.91 | 35.23 |  |

Abbreviations: SBP, systolic blood pressure ; DBP, diastolic blood pressure; NS, not stated; HWE, Hardy–Weinberg equilibrium.

^*^ The P-value of HWE determined by the χ^2^ test.
